# Impact of radiographer immediate reporting of X-rays of the chest from general practice on the lung cancer pathway (radioX): a randomised controlled trial

**DOI:** 10.1136/thorax-2022-219210

**Published:** 2022-11-08

**Authors:** Nick Woznitza, Bhagabati Ghimire, Anand Devaraj, Sam M Janes, Keith Piper, Susan Rowe, Angshu Bhowmik, Natasha Hayes, Daniel Togher, Nikita Arumalla, Erik Skyllberg, Iain T H Au-Yong, Susan Geary, Bindu George, Sarah Sheard, Stephen Ellis, Zoheb Shah, Sue Maughn, Stephen W Duffy, David Baldwin

**Affiliations:** 1 School of Allied and Public Health Professions, Canterbury Christ Church University, Canterbury, UK; 2 Radiology Department, Homerton University Hospital NHS Foundation Trust, London, UK; 3 Radiology, University College London Hospitals NHS Foundation Trust, London, UK; 4 College of Health, Medicine and Life Sciences, Brunel University London, Uxbridge, UK; 5 Radiology, Royal Brompton Hospital, London, UK; 6 National Heart and Lung Institute, Imperial College London, London, UK; 7 Lungs for Living Research Centre, UCL Respiratory, University College London, London, UK; 8 Respiratory Medicine, Homerton University Hospital NHS Foundation Trust, London, UK; 9 Radiology, Epsom and Saint Helier Hospital NHS Trust, London, UK; 10 Rheumatology, Guy's and St Thomas' NHS Foundation Trust, London, UK; 11 Respiratory Medicine, Barts Health NHS Trust, London, UK; 12 Radiology, Nottingham University Hospitals NHS Trust, Nottingham, UK; 13 Radiology, Sherwood Forest Hospitals NHS Foundation Trust, Sutton-In-Ashfield, UK; 14 Radiology, Imperial College Healthcare NHS Trust, London, UK; 15 Radiology, Barts Health NHS Trust, London, UK; 16 Wolfson Institute of Preventive Medicine, Queen Mary University of London Barts and The London School of Medicine and Dentistry, London, UK; 17 NHS England and NHS Improvement London, London, UK; 18 School of Medicine, University of Nottingham, Nottingham, UK

**Keywords:** Lung Cancer, Imaging/CT MRI etc

## Abstract

The National Optimal Lung Cancer Pathway recommends rapid progression from abnormal chest X-rays (CXRs) to CT. The impact of the more rapid reporting on the whole pathway is unknown. The aim of this study was to determine the impact of immediate reporting of CXRs requested by primary care by radiographers on the time to diagnosis of lung cancer.

**Method:**

People referred for CXR from primary care to a single acute district general hospital in London attended sessions that were prerandomised to either immediate radiographer (IR) reporting or standard radiographer (SR) reporting within 24 hours. CXRs were subsequently reported by radiologists blind to the radiographer reports to test the reliability of the radiographer report. Radiographer and local radiologist discordant cases were reviewed by thoracic radiologists, blinded to reporter.

**Results:**

8682 CXRs were performed between 21 June 2017 and 4 August 2018, 4096 (47.2%) for IR and 4586 (52.8%) for SR. Lung cancer was diagnosed in 49, with 27 (55.1%) for IR. The median time from CXR to diagnosis of lung cancer for IR was 32 days (IQR 19, 70) compared with 63 days (IQR 29, 78) for SR (p=0.03).

8258 CXRs (95.1%) were reported by both radiographers and local radiologists. In the 1361 (16.5%) with discordance, the reviewing thoracic radiologists were equally likely to agree with local radiologist and radiographer reports.

**Conclusions:**

Immediate reporting of CXRs from primary care reduces time to diagnosis of lung cancer by half, likely due to rapid progress to CT. Radiographer reports are comparable to local radiologist reports for accuracy.

**Trial registration:**

International Standard Randomised Controlled Trial Number ISRCTN21818068. Registered on 20 June 2017.

**WHAT IS ALREADY KNOWN ON THIS TOPIC**

- Lung cancer often has worse outcomes when compared with other tumour sites. A chronic shortage of consultant radiologists and suboptimal logistics means that significant delays exist in the reporting of radiology examinations and referral for further investigation, consequently there is opportunity to shorten the time to diagnosis.

**WHAT THIS STUDY ADDS**

- Immediate reporting by radiographers significantly shortened the time to diagnosis by a median of 31 days, half that seen in the control arm. Thus, implementing the minimum waiting time as recommended in the UK National Optimal Lung Cancer Pathway (NOLCP) (chest X-ray (CXR) reported before patient leaves the department) has a disproportionate impact on waiting time.

**HOW THIS STUDY MIGHT AFFECT RESEARCH, PRACTICE OR POLICY**

- Implementation of the English NOLCP reduces time to diagnosis of lung cancer. CXR reporting by radiographers can be used to create additional diagnostic capacity safely. This research supports a change in service delivery to support implementation of the NOLCP.

## Introduction

Most people who develop lung cancer have a short survival time, with 1-year survival rates of only 35%–40% in the UK, lagging behind other equivalent healthcare systems.^1^ This is influenced by the lead time to presentation and because people often present late, they can deteriorate quickly once referred with symptoms, becoming ineligible for treatment. There is randomised controlled trial evidence that rapid diagnosis can improve outcomes.^2^ Rapid diagnosis and treatment is therefore an important priority to improve outcomes, although data show consistent non-compliance with waiting times targets in the UK.^3^ The National Optimal Lung Cancer Pathway (NOLCP) is designed to accelerate the whole of the lung cancer pathway and one important feature is thatchest X-rays (CXRs) are recommended to be reported within 24 hours of acquisition and preferably before the patient leaves the radiology department so that, if indicated, a CT can be completed either the same day or within a maximum of 72 hours.^4^ Achieving this presents a logistical challenge particularly because there is a national shortage of consultant radiologists. A possible solution is to use reporting radiographers to provide additional capacity to facilitate rapid decision-making and referral for patients with suspected lung cancer. Rapid radiology review and appropriate referral should be available to all patients, including those not yet on an urgent cancer pathway. Indeed, the process for rapid reporting recommended by NOLCP has the potential to remove some patients from the urgent referral system, and thereby help both reassure patients earlier and achieve the national 28-day standard for time from referral to diagnosis.^5^

Here, we report on a randomised controlled trial of immediate radiographer (IR) reporting of CXRs to assess whether this improves time to diagnosis of lung cancer without compromising accuracy of reports.

## Methods

RadioX is a randomised controlled study conducted in London at Homerton University Hospital (HUH).^6^

### Pathways

All patients over 16 years referred by their primary care doctor for a CXR attended half-day sessions that were blocked prerandomised to either IR reporting, where the reporting occurred before the patient left the department, or standard radiographer (SR) reporting within 24 hours. The radiographer report was independently authorised, with the radiographers acting as non-medical referrers and requesting the CT scan of the chest in line with local policy. This was followed later by radiologist-blinded second-read reporting to measure accuracy of the radiographer reports. All patients in the immediate arm of the study with a CXR suspicious for lung cancer were offered same day CT scan and those who declined were scheduled for another day. For this study, the time to diagnosis was measured from the date the CXR was performed to the date the diagnosis was confirmed, being the date of the diagnostic test or the date on which a clinical diagnosis was confirmed, if no pathological sample.

### External comparison

The trial pathway differed from the usual institutional practice where CXRs are reported by either a radiologist (CR) or radiographer (RR) during or after the session in which the CXR is performed but issued within 24 hours. In order to evaluate the impact of the change to radiographer first read in the standard reporting arm, we also measured time to diagnosis and referral rates at Newham University Hospital (NUH), a nearby hospital with the same usual institutional practice (ie, without immediate reporting).

### CXR reporting evaluation

CXRs in both arms were independently reported by a local (Homerton) radiologist who was blinded to the radiographer report. All reports were then reviewed by respiratory physicians who also were provided with the clinical details on the CXR request and blinded to the reporter. The two reports per patient were evaluated for agreement. The CXRs with discordant reports were then evaluated by one of eight independent thoracic radiologists with between 2 and more than 10 years’ experience as a consultant. The thoracic radiologists were blinded to the reporter and had access to previous imaging.

Thoracic radiologists also scored the reports on four attributes: observation, interpretation, further recommendations and usefulness. The scoring system is included as online supplemental material A. Scores were dichotomised into two categories of opinion of the thoracic radiologists:

10.1136/thorax-2022-219210.supp1 Supplementary data

1. Agreement or no clinical impact of any disagreement with the report.
2. Disagreement with the report and likely significant clinical impact.

### Outcomes

The primary outcome was time to lung cancer diagnosis or discharge from the lung cancer pathway. The reported primary outcome was a better end point than the planned outcome (time from performance of the CXR to treatment with intermediate time points/discharge for lung cancer) because the date of diagnosis is easier to define than a decision to treat, which is not always clear in clinical practice and may change where additional information, for example, about fitness or staging leading to a change in decision. The secondary outcomes were the measures of agreement with thoracic radiologists in attributes of the radiographer report and the number of urgent lung cancer referrals generated. Time to diagnosis and discharge and referral rates were also measured at NUH.

A health economics analysis will be reported separately.

### Statistical analysis

We originally anticipated that the intervention would confer a difference of 11 days in time to diagnosis of lung cancer, and a common SD of 14 days. For 80% power to detect this as significant (2-sided testing), we would need 54 cancers in total. This is approximately the number of lung cancers expected in a year at HUH, so the study was designed to run for a year.

The primary analysis, comparison of times to diagnosis and discharge in those with possible lung cancer, was done using the t-test. We applied the test after log-transformation, after testing for departure from normality, since we observed that the distribution of times was strongly asymmetrical. For comparison with NUH, we compared times with diagnosis and discharge for each reporting group (immediate and standard) with the overall times to diagnosis and discharge in NUH (in which all reporting was standard) using the same method (t-test after log transformation). All tests were two-sided.

The number of urgent respiratory referrals was compared between the two groups using the χ^2^ test.

Comparison of ratings of radiologist and radiographer reports by the reviewing thoracic radiologist used McNemar methods, as they involved matched pair comparison (each case being reported by both and therefore being his or her own control).^7^

### Patient and public involvement

Patients and carers were involved in all aspects of the study. Delays in diagnosis and results were identified as a priority area. Patient feedback was given on preliminary research questions and study design, including the non-recruitment study design. Preliminary results were shared and discussed with the patient panel with the priorities identified used to inform analysis.

## Results

Between 21 June 2017 and 4 August 2018, 8682 CXRs were referred from primary care. Three radiographers (two with 1 year experience, one with 7-year experience) and thirteen local radiologists (between 2 and more than 10 years’ experience) reported CXR included in the study. Of these, 4096 (47.2%) were in sessions prerandomised to IR, while 4586 (52.8%) were in sessions prerandomised to SR. A total of 49 lung cancers were diagnosed on patients with a CXR referred from primary care, 27 (55.1%) for IR and 22 (44.9%) for SR. Of the 49 cancers, 48 (98.0%) were flagged by both the radiographer and local radiologist as abnormal or referred for an urgent respiratory appointment. One lung cancer was diagnosed on a subsequent emergency attendance for upper limb deep vein thrombosis. The demographic and history details of the subjects are shown in table 1. No differences were seen in age, sex, previous imaging with CXR or CT and smoking history.

**Table 1:** Demographic and history data on the 8682 patients recruited to the trial

Variables, measures	Immediate pathway	Standard pathway
Total patients	4096	4586
Age (years)		
Median, IQR	53 (39, 65)	53 (40, 65)
Mean, SD	53 (17)	53 (17)
Sex		
Female	2214 (54.1)	2491 (54.3)
Male	1882 (45.9)	2095 (45.7)
Previous X-ray		
Yes	2297 (56.1)	2583 (56.3)
No	1799 (43.9)	2003 (43.7)
Previous CT scan		
Yes	307 (7.5)	334 (7.3)
No	3789 (92.5)	4252 (92.7)
Smoking status		
Current	555 (13.6)	650 (14.2)
Former	315 (7.7)	386 (8.4)
Never	255 (6.2)	260 (5.7)
Unknown	2971 (72.5)	3290 (71.7)

### Referrals, time to diagnosis and discharge

Table 2 shows the outcome of the CXR by randomisation group. A total of 339 urgent lung cancer referrals were made to Homerton by primary care clinicians due to abnormal imaging or high clinical risk, 150 (36.3%) in the IR group and 189 (55.8%) in the SR group. The median time from the date of the CXR to diagnosis of lung cancer was 32 days for IR (IQR 19–70) and 63 days for SR (IQR 29–78); p=0.03. The days to diagnosis of lung cancer ranged from 10 to 169 for IR and from 18 to 319 for SR. For those with an urgent cancer pathway referral but not diagnosed with lung cancer, there was no significant difference between groups with respect to time to discharge from the lung cancer pathway. The median time to discharge for IR was 30 days (IQR 17–64) and 27 days (IQR 14–16) for SR. The log-transformed times to diagnosis and discharge did not depart significantly from normality (p=0.3 and p=0.5 respectively).

**Table 2:** Referrals and outcomes for the 8682 patients in the study

Outcome	Immediate reporting	Standard reporting
Total patients	4096	4586
Lung cancer suspected		
Yes	1326 (32.4)	1511 (33.0)
No	2757 (67.3)	3062 (66.7)
Known	13 (0.3)	13 (0.3)
2ww referral		
Yes	150 (3.7)	189 (4.1)
No	3946 (96.3)	4397 (95.9)
Total cancers diagnosed (%)	27 (0.7)	22 (0.5)
Cancer diagnosis days		
Median, IQR	32 (19, 70)	63 (29, 78)*
Mean (SD)	47.2 (35.8)	81.6 (78.5)
Discharge days (no lung cancer)		
Median (IQR)	30 (17, 64)	27 (14, 61)
Mean (SD)	54.4 (60.4)	50.3 (63.7)

*P=0.03.

In the immediate group, 42 (1.0%) patients were referred for an urgent, non-cancer respiratory appointment compared with 72 (1.6%) in the control group; p=0.03. Of the lung cancers diagnosed, all had a CT scan although for 2 patients this was more than 6 months later; 15 of the patients with lung cancer had same day CT scan.

### CXR reporting evaluation

For IR, the median (IQR) report turnaround time (TAT) was zero hours (0–1) and was achieved for 71% of all reports (range 0–200 hours). Median SR TAT was 1 hour (IQR 0–2) with 99% of CXRs reported in less than 24 hours (range 0–504 hours). Overall, 8258 CXRs (95.1%) were reported by both radiographers and local radiologists, the remaining 424 CXRs had only a radiographer report. Discordant reports were identified by respiratory physicians in 1361 cases and all CXRs were reviewed by independent thoracic radiologists. Table 3 shows whether the reviewing thoracic radiologist agreed with radiographer and local radiologist reports independently, but as noted above, the statistical significance testing and confidence intervals on differences were carried out using McNemar methods. A positive difference in agreement rates in the table implies that the thoracic radiologist agreed more often with the radiologist’s report and a negative value indicates that the thoracic radiologist agreed more often with the local radiologist’s report. Ratings of radiographer and radiologist reports were very similar. The only significant difference was for the usefulness of the report, for which the reviewer found the radiographer’s report useful in 1250 (91.8%) and the radiologist useful in 1196 (87.9%), a difference of 3.9% (95% CI: 1.7 to 6.3%; p<0.001).

**Table 3:** Results of thoracic radiologists’ reviews of radiographers’ and radiologists, reports, in 1361 cases where these differed

Attribute of report	Review finding	Radiographer report (%)	Radiologist report (%)	Difference (CI)	Significance
Observation	Agree	1133 (83.2)	1145 (84.1)	−0.9% (−3.8 to 2.0)	p=0.5
	Disagree	228 (16.8)	216 (15.9)		
Interpretation	Agree	1119 (82.2)	1138 (83.6)	−1.4% (−4.3 to 1.5)	p=0.3
	Disagree	242 (17.8)	223 (16.4)		
Further recommendations	Agree	1023 (75.2)	986 (72.4)	2.8% (−0.8 to 6.3)	p=0.1
	Disagree	338 (24.8)	375 (27.6)		
Usefulness	Agree	1250 (91.8)	1196 (87.9)	3.9% (1.6 to 6.3)	p<0.001
	Disagree	111 (8.2)	165 (12.1)		
Accuracy	Agree	839 (61.6)	831 (61.1)	0.6% (−3.6 to 4.8)	p=0.8
	Disagree	522 (38.4)	530 (38.9)		

A positive difference in agreement rates in the table implies that the thoracic radiologist agreed more often with the radiographer’s report and a negative value indicates that the thoracic radiologist agreed more often with the radiologist’s report.

### External comparison

In NUH in the same year, there were 11 lung cancers diagnosed, and the median time from CXR to diagnosis was 63 (IQR 39–90). The time to diagnosis in HUH was significantly shorter (p=0.02) than the time to diagnosis in NUH, in the immediate group but not in the standard reporting group (p=0.7). In NUH for those referred to the lung cancer pathway but not diagnosed with lung cancer, the median time to discharge was 42 days (IQR 24–71). The time to discharge was significantly shorter in Homerton for both the immediate (p=0.04) and standard (p=0.0002) reporting groups.

## Discussion

To our knowledge, this is the first randomised study to test a specific element of the English NOLCP, now a major priority for improvement of service for people with suspected lung cancer in the UK.^4^ We found that IR of CXRs from primary care substantially reduced the time to diagnosis of lung cancer, reducing it by half. As part of a parallel, non-randomised study, we also compared time to diagnosis with that of a neighbouring general hospital and found similar time to diagnosis as the SR arm. There were significantly fewer urgent referrals to respiratory services in the IR group. There was no difference in time to discharge from the pathway for urgent suspected lung cancer referrals without lung cancer. Thoracic radiologist review of radiographer and radiologist reports did not reveal any relevant differences. Specialist thoracic radiologists judged only 61% of discordant case reports to be accurate for both radiographers and radiologists. While this may seem low, table 3 suggests that this is due to a combination of three other measures (observation, interpretation and recommendations) that may have combined to produce such a low figure.

### Strengths and weaknesses

The major strength of the study is that it is a randomised controlled trial with block randomisation by session to minimise the chance of a change of practice during the study (in the control arm). The limitations are the relatively small numbers of lung cancers (49) and the fact that the trial took place in a single institution. However, the number was close to that anticipated in the sample size calculations, and both the magnitude of the observed difference and the statistical significance place confidence in the primary outcome. Furthermore, our findings were supported by the external comparison with a neighbouring hospital using the standard reporting pathway, where the median waiting time for lung cancer diagnosis was identical to the median in our control group, giving some support to the potential generalisability of the results.

Concern about the accuracy of radiographer reports has been partly addressed in this study, and the only cancer missed was not seen by either radiographer or local radiologist. However, a much larger sample may be required to measure any smaller but still clinically important differences. More important may be the training and continued professional development to monitor and maintain accuracy if radiographers are employed in this way.

### Other work in this area

Evison *et al* reported a service improvement project focused on the time from urgent lung cancer referral to CT scan of the chest and immediate report, reducing the median time for a CT scan report of the chest and discharge for non-cancer diagnoses from 3 days to same day.^8^ The Manchester RAPID programme shows promising results but was not a randomised controlled trial. It does, however, show the importance of rapid logistics in the lung cancer pathway and supports the findings of our trial. Implementation of immediate CXR reporting and same day triage of CT scan of the chest, as a preferred option in the NOLCP is likely to significantly reduce the time to diagnosis for patients with lung cancer.

Malalasekera *et al* found that long waiting times for diagnostic tests were a frequent source of delayed diagnosis of lung cancer, occurring in 106 of 136 cases.^9^ This is a common finding in the English National waiting time monitoring.^3^ Ellis and Vandermeer reported a median of 22 days (IQR 0–38 days) from first presentation to last test ordered by primary care and a further 23.5 days (IQR 10–56 days) from first specialist appointment to last diagnostic test.^10^ Some cancers may also progress during the diagnostic period, emphasising the need for rapid diagnosis to improve outcomes.^11^

## Conclusion

The English NOLCP gives the preferred option of immediate reporting of CXRs before the patient leaves the department to improve logistics. Given the national shortage of radiologists, radiographer reporting is a way to achieve this. We have confirmed, in a randomised controlled trial, that this strategy does indeed have the anticipated favourable impact on logistics, which translates into a substantial reduction in time to diagnosis. It is thus recommended that this be considered by clinical teams to provide patients with faster diagnosis.

## Data Availability

Data are available upon reasonable request.
