# Supplementary material for: Impact of radiographer immediate reporting of X-rays of the chest from general practice on the lung cancer pathway (radioX): a randomised controlled trial
Source: Thorax. 2022 Nov 8;78(9):890–4. doi: 10.1136/thorax-2022-219210 (PMC10447363; doi:10.1136/thorax-2022-219210)
Supplement: Supplementary data [file thorax-2022-219210supp001.pdf]

Supplementary Material A. Scoring criteria used by thoracic radiologists when reviewing discordant CXR reports

Observational (including observations that have been reported, and observations that have not been reported but should have been) [1 and 2 agree, 3 disagree].
1 Agree completely with observations
2 Minor observational error not likely to be clinically significant
3 Major observational error, likely to have some clinical impact

Interpretation. [1 and 2 agree, 3 disagree].
1 Agree with interpretation of findings
2 Disagree with interpretation, but clinical impact likely to be negligible
3 Disagree with interpretation, likely to be significant clinical impact

Further recommendations. [1 and 2 agree, 3 and 4 disagree].
1 Agree with further recommendations (Also if no recommendations are made and are not required, this score applies)
2 Disagree with further recommendations, but of no clinical impact
3. Agree that recommendations are required, but disagree with completeness of recommendation. Potential for some clinical impact.
4 Disagree with further recommendations and likely to have clinical impact. Also if recommendations should have been made and are not reported, this score would also apply.

Usefulness of report. [1 and 2 agree, 3 and 4 disagree].
1 Clear and actionable report, (this is based purely on the layout and language of the report, not on accuracy which is capture in scores above)
2: Minor areas of lack of clarity but very unlikely to lead to clinical impact.
3: Lack of clarity could be misinterpreted or not understood by GP and potential for clinical impact
4: Significant lack of clarity in report, with high likelihood for misunderstanding and clinical impact. This could apply to the entire report or just one component of the report if it has potential for clinical impact.

Report Accuracy
1 Both Correct - No Significant Discrepancy
2 Report A correct
3 Report B correct
4 Neither correct

Woznitza N, *et al. Thorax* 2023; 78:890–894. doi: 10.1136/thorax-2022-219210
